# Comparison of noise-normalized minimum norm estimates for MEG analysis using multiple resolution metrics

**DOI:** 10.1016/j.neuroimage.2010.09.053

**Published:** 2011-02-01

**Authors:** Olaf Hauk, Daniel G. Wakeman, Richard Henson

**Affiliations:** Cognition and Brain Sciences Unit, Medical Research Council, 15 Chaucer Road, Cambridge CB2 7EF, UK

**Keywords:** Source analysis, Inverse problem, Localization error, Spatial dispersion, Amplitude

## Abstract

Noise-normalization has been shown to partly compensate for the localization bias towards superficial sources in minimum norm estimation. However, it has been argued that in order to make inferences for the case of multiple sources, localization properties alone are insufficient. Instead, multiple measures of resolution should be applied to both point-spread and cross-talk functions (PSFs and CTFs). Here, we demonstrate that noise-normalization affects the shapes of PSFs, but not of CTFs. We evaluated PSFs and CTFs for the MNE, dSPM and sLORETA inverse operators, on the metrics dipole localization error (DLE), spatial dispersion (SD) and overall amplitude (OA). We used 306-channel MEG configurations obtained from 17 subjects in a real experiment, including individual noise covariance matrices and head geometries. We confirmed that for PSFs DLE improved after noise normalization, and is zero for sLORETA. However, SD was generally lower for the unnormalized MNE. OA distributions were similar for all three methods, indicating that all three methods may greatly underestimate some sources relative to others. The reliability of differences between methods across subjects was demonstrated using distributions of standard deviations and *p*-values from paired *t*-tests. As predicted, the shapes of CTFs were the same for all methods, reflecting the general resolution limits of the inverse problem. This means that noise-normalization is of no consequence where linear estimation procedures are used as “spatial filters.” While low DLE is advantageous for the localization of a single source, or possibly a few spatially distinct sources, the benefit for the case of complex source distributions is not obvious. We suggest that software packages for source estimation should include comprehensive tools for evaluating the performance of different methods.

## Research Highlights

►Noise-normalization of inverse estimators affects the shapes of PSFs, but not CTFs. ►Spatial resolution of EEG/MEG should be assessed using multiple resolution metrics. ►Resolution metrics should describe localization, spatial extent and amplitude. ►dSPM and sLORETA have lower localization error than MNE, but larger spatial extent. ►The benefit of dSPM/sLORETA over MNE for complex source distributions is not clear.

## Introduction

The ultimate goal of neuroimaging is to determine the accurate spatio-temporal dynamics of perceptual and cognitive processes in the human brain. There exists a well-known “dichotomy” between metabolism-based methods such as functional magnetic resonance imaging (fMRI) and positron emission tomography (PET) with spatial resolution on the millimetre scale on the one hand, and electrophysiological methods such as electro- and magnetoencephalography (EEG and MEG) with millisecond temporal resolution on the other (e.g. Dale and Halgren, 2001). The temporal resolution of fMRI and PET is generally accepted to be fundamentally limited by the hemodynamic response function. The spatial resolution of EEG and MEG, however, is still a matter of debate. The non-uniqueness of the associated inverse problem allows infinitely many solutions for any given data set, and consequently a large number of different methods have been suggested (e.g. Baillet et al., 2001).

Distributed source solutions are an important class of methods, because they rely on minimal modelling assumptions. They are therefore applicable to complex data sets or at high noise levels (Dale and Sereno, 1993; Greenblatt et al., 2005; Hämäläinen and Ilmoniemi, 1984). In particular, methods of the minimum norm type are widely used and implemented in most software packages. “Classical” least-squares minimum norm estimation (MNE) was introduced to MEG analysis by Hämäläinen and Ilmoniemi (1984). However, this method has the undesirable property that maxima of inverse solutions are biased towards the sensors (e.g. Fuchs et al., 1999; Lin et al., 2006). Several different approaches have been suggested to alleviate this problem.

(1) Depth weighting. Forward solutions for all sources in the model are normalized by a measure of their overall amplitude (e.g. a norm of the corresponding columns of the leadfield matrix) (e.g. Fuchs et al., 1999; Lin et al., 2006), which has been shown to improve localization error (Lin et al., 2006).
2) Noise normalization. The estimated current at each source location is divided by an estimate of the noise at that location, which can be obtained by applying the inverse operator to the signal covariance matrix, as in dynamic statistical parametric mapping (“dSPM”) (Dale et al., 2000), or using the diagonals of the model resolution matrix, as in standardized low-resolution electromagnetic tomography (sLORETA) (Pascual-Marqui, 2002).

dSPM and sLORETA both implicitly perform “depth weighting” as well—sources with generally higher amplitude will be normalized by higher noise levels or source variances. In the following, we will therefore only compare the classical MNE, dSPM and sLORETA. sLORETA is a particularly interesting candidate because it is designed to have zero dipole-localization error, i.e. for every point source, sLORETA produces a PSF whose maximum is exactly at the correct location (Pascual-Marqui, 2002). Although this is clearly an advantageous property, it has been pointed out that this property alone is not a sufficient criterion for comparing different methods—in fact, even “trivial” methods with otherwise very undesirable properties can show zero dipole localization error (Grave de Peralta et al., 2009).

This raises the question of which metrics one should use to fairly evaluate and compare distributed inverse solutions. Several studies have used different measures of localization error and spatial extent (Fuchs et al., 1999; Grave de Peralta-Menendez and Gonzalez-Andino, 1998; Molins et al., 2008). A recent study by Molins et al. (2008) evaluated resolution for MNE on several different MEG and EEG sensor configurations. They defined three measures that described different aspects of PSF distributions: (1) Dipole Localization Error (DLE), i.e. the distance of a solution's peak to the true location of a point source; (2) Spatial Dispersion (SD), i.e. a measure of the “width” of the distribution around the true source location; (3) the Resolution Index (RI), reflecting how much the activity at a particular location contributes to the amplitude estimate for that location. These measures were computed for every point source in the model, and could therefore be visualized as distributions across the whole cortical surface. Their simulations were performed at realistic noise-levels obtained from a previous experiment, and included the noise covariance matrix in the computation of the inverse operator.

Following this and other previous evaluation studies (Fuchs et al., 1999; Menendez et al., 1996; Molins et al., 2008), we will consider three “categories of resolution” in our simulations:

(1) Localization error, i.e. the distance between the location inferred from the estimated solution and the true source location.
2) Spatial extension, in analogy to the “width” of a peak-shaped distribution.
(3) Amplitude estimation, i.e. how amplitudes of PSFs/CTFs differ relative to each other.

**Fig. 1:**
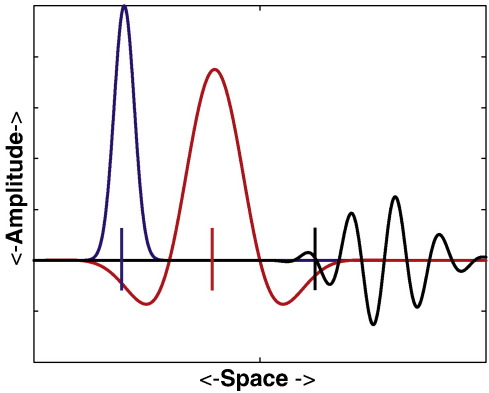
Illustration of possible deviations from the ideal case of point-spread functions (PSFs) and cross-talk functions (CTFs). The curves are schematic PSFs/CTFs for target locations indicated by colored vertical lines. The blue curve illustrates the almost-ideal case, with a symmetric peak centered on the target location. The red curve illustrates the case of a well-centered PSF or CTF, but with a broader peak and sidelobes. The black curve represents the worst case where PSFs/CTFs are not well centered, not bell-shaped and exhibit large sidelobes.

The RI measure of Molins et al. (2008) included both amplitude and localization error. We decided to use a measure of overall amplitude, because relative amplitudes of different PSFs are important in the case of simultaneously active sources. The aspects of resolution captured by these metrics are illustrated in Fig. 1.

In addition to the question of “how” to evaluate distributed methods, another important question is “what” to evaluate. Most of the earlier studies focused on localization of point sources, using PSFs (Fuchs et al., 1999; Hämäläinen and Ilmoniemi, 1984; Ioannides et al., 1993; Pascual-Marqui et al., 1994; Sekihara et al., 2005). PSFs describe how activity from one point source would project to other locations if it were active in isolation. This information is valuable if we expect a single or only few clearly separable sources. However, if we expect multiple sources or complex source patterns, the question “how is a current estimate at one location (e.g. in a region of interest) affected by sources around it?” becomes more relevant. This information is provided by CTFs, and has recently gained momentum in the area of EEG/MEG source estimation (Grave de Peralta Menendez et al., 1997a; Hauk, 2004; Liu et al., 2002; Lütkenhöner and Grave de Peralta Menendez, 1997; Molins et al., 2008).

As we will show below theoretically and in simulations, all normalization procedures mentioned above affect the shapes of PSFs, but only the overall amplitudes of CTFs. Therefore, they may affect the localization properties for point sources, but not the spatial filter properties of estimators for different regions of interest. In our simulation study, we computed PSFs and CTFs for a realistic 306-channel MEG set-up. We applied three measures for the resolution categories localization error, spatial extent and amplitude estimation, and used them to compare unnormalized MNE, dSPM and sLORETA. The aim was to investigate whether a general increase of spatial resolution can be achieved, or whether improvement in one measure of resolution may lead to deterioration in another. The results will be discussed with respect to their relevance for different applications of distributed source analysis.

## Theory and methods

### General

In matrix notation, estimating the distribution of current densities described by a vector **j** means solving the underdetermined linear equation

(1) d=Lj

where **d** is the data vector, **L** the leadfield matrix (or forward solution), and **j** the source current density vector (e.g. Hämäläinen et al., 1993; Sarvas, 1987). While the data vector **d** is naturally discrete, since measurements are taken at discrete locations in space (~ several tens or hundreds), the source current vector **j** is an approximation of a naturally continuous current distribution. In distributed source estimation, **j** typically contains by far more elements than there are sensors. As a consequence, there are distributions $j_{0}\neq 0$ that do not produce any measurable signals, i.e. for which $Lj_{0}=0$, which constitutes the “non-uniqueness” of the underdetermined inverse problem (e.g. Bertero et al., 1985; Golub and van Loan, 1996). All we can hope for is an estimate $\hat{j}$ that comes as close as possible to the real but unknown current distribution **j**, given the data and possibly further a priori information.

Linear estimation methods attempt to find such an estimate by multiplying the data by an inverse operator matrix **G** (e.g. Menke, 1989):

(2) $$\hat{j}=Gd$$

By inserting Eqs. (1) into (2) we obtain a simple relationship between the true and the estimated current distribution:

(3) $$\hat{j}=Gd=GLj=Rj$$

This defines the “resolution matrix” **R**, which plays a key role in describing and evaluating the resolution properties of linear estimators (e.g. Backus and Gilbert, 1968; Menke, 1989), and has been applied to EEG and MEG data in several previous studies (e.g. Grave de Peralta Menendez et al., 1997b; Liu et al., 2002; Molins et al., 2008).

The resolution matrix can already answer two important questions about linear inverse estimators:

(1) How is a point source represented in the estimated solution, i.e. how is it distorted by the inverse estimator?
(2) How does a point source in one location affect the amplitude estimation for a source in another location?

Question 1 can be answered by looking at the columns of **R,** which represent the point spread functions (PSFs) of the inverse estimator **G**. Note that due to the linearity of the problem, the superposition principle holds: The solution for multiple sources is the sum of the solutions for the individual sources. Question 2 can be addressed by looking at the rows of **R**, i.e. the cross-talk functions (CTFs) of **G**. Note that because of Eq. (3), these are necessarily linear combinations of the rows of the leadfield matrix **L**.

### MNE, dSPM, sLORETA

A common expression for the classical MNE is

(4) $$G_{\text{MNE}}=L^{T}{\left(LL^{T}+\lambda C\right)}^{-1}$$

where *λ* is the regularization parameter and C the noise covariance matrix. The resolution matrix in this case is

(5) $$R_{\text{MNE}}=L^{T}{\left(LL^{T}+\lambda C\right)}^{-1}L$$

which is a symmetric matrix, and therefore PSFs and CTFs for elements *i* are the same.

Both dSPM and sLORETA are derived from $G_{\text{MNE}}$ by normalizing its rows, which can be formulated as multiplying $G_{\text{MNE}}$ by a diagonal matrix W from the left:

(6) $$G_{\text{dSPM}}=W_{\text{dSPM}}G_{\text{MNE}}$$

(7) $$G_{\text{sLOR}}=W_{\text{sLOR}}G_{\text{MNE}}$$

This yields the resolution matrices

(8) $$R_{\text{dSPM}}=W_{\text{dSPM}}R_{\text{MNE}}$$

(9) $$R_{\text{sLOR}}=W_{\text{sLOR}}R_{\text{MNE}}$$

Because the W matrices are diagonal, each row *i* of the MNE resolution matrix is scaled by the factor *W*_*ii*_. As a consequence, the shapes of the CTFs (rows of **R**) do not change. Only the shapes of PSFs (columns of **R**), and therefore potentially locations of peaks and their spatial extensions, are affected by this normalization procedure.

For dSPM, the normalization matrix contains the minimum norm estimates of the noise at each source (Lin et al., 2006), derived from the noise covariance matrix, i.e.

(10) $$W_{\text{dSPM}}^{2}=\text{diag}\left(G_{\text{MNE}}^{}CG_{\text{MNE}}^{\text{T}}\right)$$

For sLORETA, the normalization uses the diagonal of the MNE resolution matrix $R_{\mathrm{MNE}}$ (Pascual-Marqui, 2002):

(11) $$W_{\text{sLOR}}^{2}\text{= diag}\left(R_{\text{MNE}}^{}\right)=\text{diag}\left(G_{\text{MNE}}^{}L\right)=\text{diag}\left(G_{\text{MNE}}^{}\left(LL_{}^{\text{T}}+C\right)G_{\text{MNE}}^{\text{T}}\right)$$

It has been shown that this sLORETA type of normalization guarantees that the PSF of a source *i* assumes its maximum at element *i*. In other words, it has zero dipole localization error (Pascual-Marqui, 2002). However, it does not allow similar conclusions about other aspects of PSFs (e.g. spatial extent, local extrema, etc.), or about the shape of CTFs.

### Simulations

Simulations were carried out on 17 data sets from a real MEG experiment. The MEG system was an Elekta Neuromag Vectorview, which contains 102 magnetometers and 204 planar gradiometers (Elekta AB, Stockholm, Sweden). The noise covariance matrices for each data set were computed concatenating baseline intervals of 200 ms duration before the 146 stimuli that were presented during the experimental session (line drawings in a picture naming task, which are of no relevance to the present study). For regularization, the default signal-to-noise ratio in the MNE software was used (SNR = 3). For dSPM computation, the number of averages was set to 100. MEG sensor configurations and MRI images were coregistered based on the matching of about 50–100 digitized locations on the scalp surface with the reconstructed scalp surface from the FreeSurfer software (see below).

High-resolution structural T1-weighted MRI images were acquired in a 3 T Siemens Tim Trio scanner at the CBU using a 3D MPRAGE sequence, field-of-view 256 mm × 240 mm × 160 mm, 1 mm isotropic resolution, TR = 2250 ms, TI = 900 ms, TE = 2.99 ms, flip angle 9 degrees. Structural MRI images were processed using automated segmentation algorithms of the FreeSurfer software (Version 4.3; http://surfer.nmr.mgh.harvard.edu/) (Dale et al., 1999; Fischl et al., 2001, 1999).

The result of the FreeSurfer segmentation was processed further using the MNE software package (Version 2.6; http://www.nmr.mgh.harvard.edu/martinos/userInfo/data/sofMNE.php). The original triangulated cortical surface (consisting of several hundred thousand vertices) was downsampled to a grid using the traditional method for cortical surface decimation with an average distance between vertices of 5 mm, which resulted in approximately 10,000 vertices. A boundary element model (BEM) containing 5120 triangles was created from the inner skull surface, which was created using a watershed algorithm. Dipole sources were assumed to be perpendicular to the cortical surface. Therefore, no “depth weighting” of the leadfield was applied, since for fixed orientations this would result in high weightings for both deep sources and superficial radial sources.

PSFs and CTFs for MNE, dSPM and sLORETA were computed in the MNE software and using the MNE Matlab toolbox (Version 2.6). The evaluation metrics applied to each individual PSF and CTF are listed in Table 1. They were chosen to capture aspects of mislocalization, spatial extent and relative amplitude as illustrated in Fig. 1. Dipole localization error (DLE) is the most widely used metric for localization accuracy, because inferences about localization in real data sets are usually made on the basis of peaks of activation. As a metric for spatial extent, we used the spatial dispersion (SD) used in Molins et al. (2008) for comparison. A larger value on this measure is indicative of a more widely distributed PSF or CTF. The overall amplitude (OA) of PSFs and CTFs was assessed using the sum of absolute values of amplitudes at vertices across the whole source space. While for DLE and SD the absolute values are of interest, for OA relative differences between PSFs/CTFs are more relevant—i.e. whether some sources are largely overestimated with respect to other sources, and could therefore obscure them when active simultaneously. We therefore normalized the distributions of overall amplitude to their maxima for each individual data set before grand-averaging. This normalization procedure removed inter-individual amplitude differences, e.g., due to sensor positioning, signal-to-noise ratio, etc.

These metrics were computed for all PSFs/CTFs at all vertices in the source space for each subject. The result for each individual was then morphed to the average brain across all subjects, using the spherical morphing procedure in the FreeSurfer software. A grand-average of the morphed surfaces was computed, which was then displayed on the inflated average cortical surface.

## Results

**Fig. 2:**
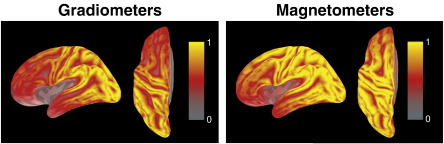
Grand-average sensitivity maps (sum of squares for each column of the leadfield) for gradiometers and magnetometers, normalized to their maxima. Lateral and top views of the left hemisphere are shown. Sources were assumed to have fixed orientation perpendicular to the cortical surface.

Fig. 2 presents the grand-average sensitivity maps for the left lateral and top views for our simulation configuration. For each location in source space, the sum of squares of the corresponding column of the forward solution was computed, and plotted as a distribution. Our sensor configuration is most sensitive to sources close to the gyri, as is generally the case for MEG (Goldenholz et al., 2009; Hämäläinen et al., 1993; Hillebrand and Barnes, 2002). Sensitivity dramatically drops off with distance from the sensors, resulting in low sensitivity within the Sylvian fissure and in orbito-frontal cortex. The inferior sensitivity to frontal compared to posterior sources is most likely due to the fact that our participants were seated with the backs of their heads against the back of the dewar, i.e. the distance between frontal sources and the sensors was larger than for posterior sources.

**Fig. 3:**
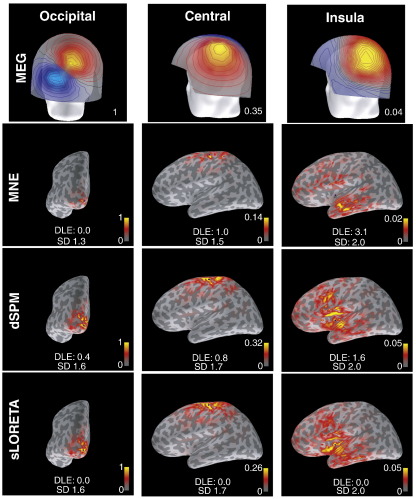
Magnetometer topographies (top row) and PSFs for 3 selected locations (columns) for all 3 methods (bottom rows). Simulations were carried out for one data set. Target locations are indicated by small blue dots. Dipole Localization Error (DLE) is indicated in centimeters, Spatial Dispersion in square-root-of-centimeters. All images are scaled to their individual maximum. The values at the top of the scale bars indicate relative maximum amplitudes with respect to the left-most column. Note that CTFs for these target locations would be identical to the corresponding PSFs for MNE for all three methods.

Fig. 3 presents PSFs for 3 sources at different locations for MNE, dSPM and sLORETA. This is meant to illustrate the main properties of these methods, rather than to provide a systematic comparison. The locations were chosen in order to represent sources at the tip of the occipital lobe, at the top of the central sulcus, and a deeper source in the insula. This figure also contains the corresponding values for DLE, SD, as well as relative overall amplitude. The source in the occipital lobe is well localized by all three methods. However, the MNE distribution is noticeably less extended than those for dSPM and sLORETA, which are similar to each other. A similar pattern of results is obtained for the source in the central sulcus, although MNE is more spread out than for the occipital source. Again, dSPM and sLORETA are more extended and resemble each other (though note that DLE for sLORETA is zero, as expected). For the source in the insula, MNE produces maximum activation in anterior middle temporal areas and negligible activation around the true source location, i.e. activation is clearly mislocalized (by about 3 cm), being projected to areas closer to the sensors. Both dSPM and sLORETA show maximum activation around the true source location. However, activation with comparable amplitude also spreads to inferior frontal and superior temporal areas, with some noticeable activation also in inferior temporal regions. Note also that the relative OA between the insula and occipital location is very low for each method (i.e., a factor of 20–50).

**Fig. 4:**
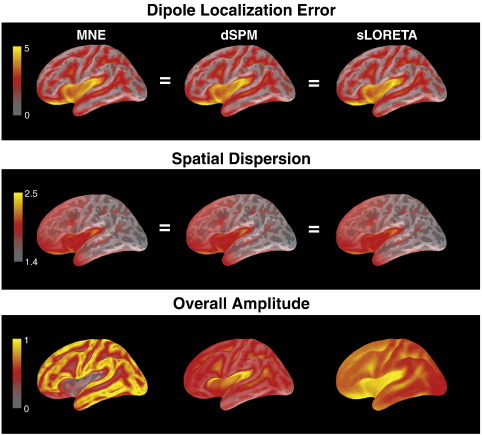
Resolution metrics for CTFs for different noise-normalizations. Different color schemes were chosen for clearer visualization. Note that the scale for SD does not begin at zero. OA distributions were normalized to their individual maximum before grand-averaging (because only differences between locations are relevant).

Fig. 4 shows the resolution metrics dipole localization error (DLE), spatial dispersion (SD) and overall amplitude (OA) for CTFs for MNE, dSPM and sLORETA. As is obvious from the theoretical description of these methods, their DLE and SD distributions are identical, reflecting the fact that their CTFs only differ with respect to scaling, but not with respect to shape. Not surprisingly, DLE is largest within sulci, in particular in the Sylvian fissure and in orbito-frontal areas (where it is larger than 5 cm), i.e. areas that are furthest from the sensors. SD shows a similar pattern, and also exhibits larger values in anterior compared to posterior areas, resembling the sensitivity maps in Fig. 2. OA distributions differ between methods. MNE shows the greatest differences in amplitude between CTFs, with lowest amplitudes in sulci, particularly in the Sylvian fissure. Relative differences in amplitude between CTFs are smaller for dSPM and sLORETA. These methods show a somewhat reversed pattern compared to MNE, i.e. larger amplitudes for locations in sulci. This means that the amplitude estimate for the noise is lower than the amplitude estimate for point sources in the sulci, therefore overcompensating for the lower amplitudes of the MNE.

**Fig. 5:**
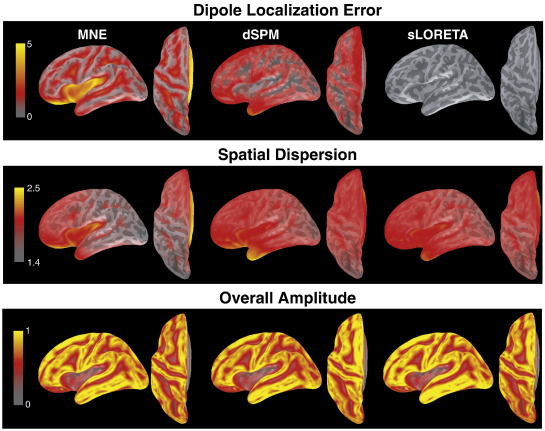
Resolution metrics for PSFs for different noise-normalizations. Different color schemes were chosen for clearer visualization. Note that the scale for SD does not begin at zero, and that the colour schemes for DLE and SD were chosen differently. OA distributions were normalized to their individual maximum before grand-averaging (only differences between locations are relevant).

**Fig. 6:**
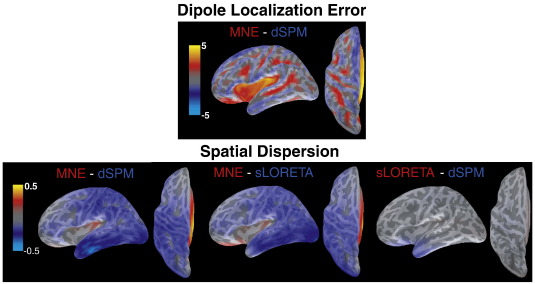
Difference distributions for DLE and SD of PSFs. Note that DLE for sLORETA is zero everywhere. DLE is indicated in centimeters, SD in square-root-of-centimeters.

Fig. 5 shows the resolution metrics applied to PSFs for all three methods. As expected, in this case shapes as well as amplitudes differ between methods. sLORETA has the theoretically predicted zero DLE at every source location. Because the resolution matrix of MNE is symmetric, the distributions for its PSFs are identical to those of its CTFs in Fig. 4. MNE shows the largest DLEs among the three methods, mainly in the Sylvian fissure and in orbito-frontal areas. While DLEs for dSPM do not reach values as high as for MNE (and in fact are close to zero in the Sylvian fissure, for example), the distribution appears to be smoother than for MNE, with larger values of DLE for dSPM close the gyri. The distributions for SD show similarities across methods, with largest values in the Sylvian fissure, the anterior temporal lobe and in orbito-frontal cortex. The differences among methods will be analyzed in more detail in Fig. 6. OA is similar among methods as well, with lowest values in sulci, the Sylvian fissure and orbito-frontal cortex. This pattern resembles the sensitivity maps in Fig. 2.

In Fig. 6, the differences among the resolution metrics applied to PSFs for different methods are shown. For DLE only the difference between MNE and dSPM is presented because the DLE for sLORETA is zero, and the subtraction would yield the same distributions as in Fig. 5. dSPM outperforms MNE on this measure in the range of several centimeters in areas that are distant from the sensors, such as the Sylvian fissure and orbito-frontal cortex, as well as in sulci. However, close to the tips of the gyri, MNE shows better performance than dSPM by about 1–2 cm.

With respect to the SD measure, MNE generally outperforms both dSPM and sLORETA, in particular in inferior temporal and occipital areas. Only in some areas of the Sylvian fissure and the orbito-frontal cortex is this pattern reversed—this is likely due to the fact that SD is confounded with DLE, i.e., a large DLE will also result in a larger SD. dSPM and sLORETA differ noticeably from each other only in the anterior temporal lobe, where sLORETA shows lower values than dSPM.

For OA, only relative differences between PSFs or CTFs are relevant. A point-by-point subtraction between different methods is therefore not informative, and the corresponding distributions are not presented.

**Fig. 7:**
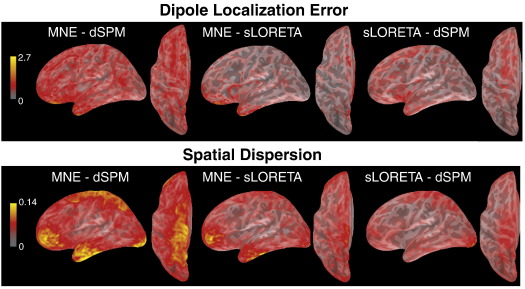
Distributions of standard deviations (across subjects) for the differences of DLE and SD, corresponding to Fig. 6.

**Fig. 8:**
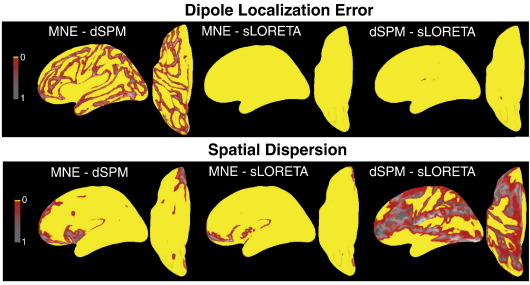
Distributions of *p*-values resulting from paired *t*-tests (across subjects) for the differences of DLE and SD, corresponding to Figs. 6 and 7. Yellow colour indicates *p*-values below 0.05.

Figs. 7 and 8 provide statistical measures for the differences presented in Fig. 6. These figures also contain the differences in DLE between sLORETA and MNE as well as dSPM, which can be interpreted as the comparisons of MNE and dSPM to zero. Fig. 7 presents the distributions of standard deviations across subjects, for the differences among MNE, dSPM and sLORETA. Fig. 8 shows the corresponding *p*-value distributions obtained by paired two-tailed *t*-tests. According to Fig. 7 there is some variability across subjects, possibly due to inter-individual differences in cortical folding (which is not the main focus of the present paper). Variability in DLE is largest between MNE and dSPM, which is not surprising given DLE for sLORETA is always zero. The same pattern is observed for SD. Fig. 8 demonstrates that the differences presented in Fig. 6 are reliable over large parts of the cortical surface. While DLE is reliably different between dSPM and sLORETA across the whole cortical surface, they are more similar to each other with respect to SD.

## Discussion

We compared three different distributed source solutions–MNE, dSPM and sLORETA–using three resolution metrics designed to evaluate localization error (DLE), spatial extension (SD), and amplitude estimation (OA). These metrics were applied to point-spread functions (PSFs) as well as cross-talk functions (CTFs). We demonstrated theoretically that noise-normalization procedures, such as dSPM and sLORETA, only affect the shapes of PSFs, but not CTFs. This was obviously reflected in our simulations, and the practical implications will be discussed below. For PSFs, the DLE for sLORETA is zero, which is the main idea behind this method (Pascual-Marqui, 2002). DLE for dSPM, although not zero, was generally lower than for MNE (Fig. 5), with the latter showing errors of more than 5 cm in deeper brain structures such as the Sylvian Fissure or orbito-frontal cortex. This reflects MNE's bias towards source locations close to the sensors. However, for the SD measure this pattern was reversed: MNE generally produced lower values than both dSPM and sLORETA. This indicates that although the maxima of MNE distributions may be mislocalized, they may still be less extended in space. The example PSFs in Fig. 3 illustrate this: MNE exhibits the most focal peaks of the three methods. For a source in the depth of the Sylvian Fissure, this peak is mislocalized by about 3 cm. However, although dSPM and sLORETA show lower DLE values, their distributions contain several local maxima (“ghost sources”) with almost peak amplitude at considerable distance from the true source location. If such a pattern were encountered for a real data set, without prior knowledge about the true number of sources, then unambiguous localization of the neural generators would be impossible. We also showed that the amplitude of estimated PSFs varies drastically with source location, in particular with source depth. This is the case for all three methods, and is also illustrated in Fig. 3. Overall amplitude for the deepest source (“Insula”) is about 1/50 of the amplitude of the occipital source for MNE, and about 1/20 for dSPM and sLORETA. In the case of multiple sources, those with large estimated amplitudes are likely to overshadow weaker ones. The reliability of differences between methods across subjects was demonstrated using distributions of standard deviations and *p*-values from paired *t*-tests (Figs. 7 and 8).

CTFs describe how much the estimate for a particular location is affected by each source in the source space (e.g. Grave de Peralta Menendez et al., 1997b; Liu et al., 2002). As has been pointed out previously (Grave de Peralta-Menendez and Gonzalez-Andino, 1998; Grave de Peralta Menendez et al., 1997b), CTFs are less “corruptible” than PSFs. While PSFs are linear combinations of the columns of the inverse operator matrix, and can therefore be “designed” by the experimenter, the CTFs are necessarily linear combinations of the leadfields, which are determined by the sensor configuration and head geometry. It is important to remember that the shape of CTFs is the same for all three methods, and that for MNE the PSFs and CTFs have the same shape, because MNE's resolution matrix is symmetric. Therefore, CTFs for all three methods have the shape of PSFs for MNE (e.g. illustrated in Fig. 4)—including the property that maxima will occur close to the sensors. Estimates for amplitudes at deeper locations will therefore always be more sensitive to superficial sources, no matter which normalization procedure is used. Estimated amplitude for a deep source may therefore reflect a large amplitude at that location, or a weaker amplitude at a more superficial location. Giving more weight to the CTF at one location (e.g. by noise-normalization) increases the likelihood that it will show activation if there is an active source at that location—however, it also increases the likelihood that it will show activation when there are active sources at other locations (e.g. where the CTF has local maxima).

The concepts of PSFs and CTFs allow different interpretations of source estimation methods. PSFs describe their localization properties, i.e. the behavior of the methods in the presence of point sources. CTFs are often associated with “spatial filters”: When the amplitude of a particular source is estimated by computing a weighted average of the measured signal across sensors, the associated CTF describes how sensitive this estimate is to all possibly active sources in the model (“virtual sensor”, e.g. Hillebrand et al., 2005; Vrba and Robinson, 2001). Linear distributed source estimation methods contain spatial filters for each source as rows of the inverse operator matrix. The fact that PSFs and CTFs are columns and rows of the resolution matrix, respectively, shows that the two concepts are interrelated. MNE minimizes the difference between the resolution matrix and the identity matrix (the ideal resolution matrix) in the least-squares sense, and therefore also yields optimal spatial filters for the case where all sources may be active simultaneously (Hauk, 2004; Menke, 1989). We showed in our theory section that noise-normalization does not affect the shape of CTFs, and therefore the CTFs for dSPM and sLORETA have the same shape as that for MNE. This is relevant for applications where the “spatial filtering” interpretation is central, such as in coherence or connectivity analysis—noise-normalization will not improve spatial selectivity of spatial filters. For example, the correlation between time courses obtained for sources at different locations should not differ between MNE, dSPM and sLORETA.

This leads to the question of which method is “best.” The answer to this question depends on one's criteria for goodness. These criteria in turn depend on the question we are trying to answer from our data. In other words, every method is best when the underlying modeling assumptions are correct for the analyzed data set, and it is the experimenter who has to decide which assumptions are justified. The methods under investigation here are commonly employed in cases where no precise modeling assumptions (e.g. about the number of sources) are available. Every source can potentially contribute to the data simultaneously with any other source. In this situation, low peak localization error alone is not a sufficient criterion (Grave de Peralta et al., 2009). Even if for a point source the peak of the estimated distribution is at the correct location, local maxima may still appear as “ghost sources,” or two distinct but simultaneously active sources may not be distinguishable because their PSFs overlap significantly. Our simulations showed that dSPM and sLORETA show lower DLE than MNE, as has been reported previously (Lin et al., 2006; Pascual-Marqui, 2002). However, this is accompanied by increased spatial extent, and as the examples in Fig. 3 suggest possibly also by larger ghost sources.

Therefore, DLE alone is not a sufficient criterion for evaluating inverse operators for the case of multiple sources. While low DLE is clearly beneficial for the localization of single sources, it is not obvious that dSPM or sLORETA are better estimators for complex source configurations than the classical MNE. We would like to point out that any particular measure of resolution that reduces a PSF or CTF to a single number will not be able to capture all their relevant properties. They can only provide estimates that may allow efficient evaluation and comparison of different methods or sensor configurations, for example. However, the most accurate description of a distribution is the distribution itself. Visualization and analysis of thousands of distributions, as would be required for an exhaustive analysis of distributed source estimation methods, is clearly impractical. The use of intuitive metrics that capture the most relevant features appears to be a promising approach to us. We would therefore like to encourage developers of software packages for source estimation to provide user-friendly tools for evaluating the spatial resolution of their methods.

## Figures and Tables

**Table 1:** Resolution metrics.

Dipole localization error	Spatial dispersion	Overall amplitude
$\begin{array}{l} \text{D}LE_{i}=\sqrt{{\left(x_{\text{p}}-x_{\text{i}}\right)}^{2}}\\ x_{\text{p}}:\text{peak coordinate}\\ x_{\text{i}}:\text{true coordinate} \end{array}$	$\begin{array}{l} {\text{SD}}_{i}=\sqrt{\frac{\sum_{j}d_{\mathrm{ij}}F_{\mathrm{ij}}^{2}}{\sum_{j}F_{\mathrm{ij}}^{2}}}\\ d_{\mathrm{ij}}:\text{distance betweenlocations }j\text{ and }i \end{array}$	$A_{i}=\sum_{j}\left|F_{\mathrm{ij}}\right|$ (normalized to maximum for each subject)

Formulas for the resolution metrics used in our simulations. *F* represents the distributions of point-spread functions (PSFs) and cross-talk functions (CTFs), respectively. | | indicates the absolute value. PSFs are the columns of the resolution matrix, representing the estimated source distribution for a single point source. CTFs are rows of the resolution matrix, describing the sensitivity of an amplitude estimator for a single point source to all other sources.
